# A simple lattice-based PKE scheme

**DOI:** 10.1186/s40064-016-3300-4

**Published:** 2016-09-21

**Authors:** Limin Zhou, Zhengming Hu, Fengju Lv

**Affiliations:** 1 Information Security Center, Beijing University of Posts and Telecommunications, Beijing, 100876 China; 2 The Seventh Middle School of Zibo, Shandong, 255499 China

**Keywords:** 06D50

## Abstract

In this paper, we first present a new lattice-based PKE scheme on SIS, proving that it achieves CPA-security under DBi-ISIS assumption. Compared to some lattice-based schemes, ours has some advantages and is quite efficient as well as great simplicity. Similarly, we give a lattice-based PKE with multiple bits which is CPA secure under DBi-ISIS assumption. We hope that our contributions help to pave the way for the development of lattice-based PKEs in the future work.

## Background

Nowadays, with the development of technologies, such as cloud computing and quantum information technology, quantum computing power becomes stronger and stronger. As a result, traditional cryptosystems, e.g number-theoretic cryptosystems, could be almost broken by quantum computers. To handle this case, lattice has come up as a powerful technique to resist quantum computers and been gradually used to construct cryptography primitives which can be against quantum attack. Maybe lattice-based cryptography will be a replacement to number-theoretic cryptography in cryptography field.

Over the last decades, lattice has emerged as a very attractive foundation for cryptography. The appeal of lattice-based primitives stems from the fact that their security can often be based on worst-case hard problems such as shortest vector problem (SVP) (Micciancio 2011; Regev 2004), closest vector problem (CVP) (Micciancio 2011; Regev 2004), approximate *the shortest independent vectors problem* SIVP (one variant of SVP) (Gentry et al. 2008; Micciancio 2011; Regev 2004) and *the SVP* GapSVP (in its decision version) (Gentry et al. 2008; Micciancio 2011; Regev 2004) to within small polynomial (in the dimension *n*) factors, because they remain secure even against quantum computers.

 On lattice, there were two basic average-case problems (Micciancio and Regev 2007) that had been shown to enjoy worst-case hardness guarantee: the learning with error (LWE) problem (Regev 2005, 2009; Applebaum et al. 2009) and the small integer solution (SIS) problem (Micciancio and Regev 2007). More recently, Regev (2005) defined the LWE problem and proved that it enjoyed similar worst-case hardness under a quantum reduction. The latter was first proposed by Ajtai (1996), who showed that it was at least as hard as approximating several worst-case lattice problems, such as the decision version of the SVP, known as GapSVP (Gentry et al. 2008) to within a polynomial factor in the lattice dimension. The SIS problem (Micciancio and Regev 2007) may be seen as a variant of subset-sum over a particular additive group. Virtually, a great deal of recent lattice-based cryptographic schemes were based directly upon the above two natural average-case problems (Micciancio and Regev 2007), such as Regev (2005), Gentry et al. (2008), Regev (2009), Applebaum et al. (2009), Lindner and Peikert (2011), Orsini and Smart (2015), Hiromasa et al. (2015), Lyubashevsky and Wichs (2015), Gentry et al. (2010), Peikert et al. (2008), Peikert (2009), Garg et al. (2013).

Compared with traditional theory-based cryptosystems, such as RSA, ECC, the lattice-based cryptographic systems mainly referred to only simple linear operation, matrix-vector multiplication, modular addition, modular multiplication of small integers. Such simple algebraic structure determined the small computational complexity, small computation operation, higher operating speed that could effectively improve data encryption and decryption speed. Such advantages made the lattice-based cryptosystems be famous. On the other hand, lattice-based cryptosystems had some disadvantages. For example, they occupied large space size, such as long public key, long secret key, large ciphertext expansion, et al, which greatly limited their practical applications. However, these defects did not limit the development of the lattice-based cryptosystems. Up to date, there existed a lot of lattice-based cryptographic schemes: Regev (2005, 2009), Gentry et al. (2008), Applebaum et al. (2009), Lindner and Peikert (2011), Orsini and Smart (2015), Hiromasa et al. (2015), Lyubashevsky and Wichs (2015), Gentry et al. (2010), Peikert et al. (2008), Peikert (2009), Garg et al. (2013) etc. In addition, in 2012, Ding and Lin (2012) first constructed a lattice-based key exchange (KE) from LWE problem and first connected KE with lattice together. Li et al. (2013) proposed two KEs from the LWE problem and the SIS problem. The works of Albrecht et al. (2016), Cheon et al. (2016) and Ducas et al. (2014) presented some problems over NTRU lattice (Hoffstein et al. 1998). Zhang et al. (2015) first proposed a KE from ideal lattice. The works of Becker et al. (2016) and Laarhoven (2015) proposed some algorithms to solve hard problems, e.g. *SVP*, which were new breakthrough on lattice. Alkim et al. (2015) and Bos et al. (2015) proposed lattice-based KEs. The works of Lindner and Peikert (2011) and Poppelmann and Guneysu (2013) proposed key encryption without key encryption mechanism on the LWE problem; but Peikert (2014) proposed lattice-based key encryption with key encryption mechanism. In 2014, Wang et al. (2014) first constructed a lattice-based KE relied on Bi-ISIS problem; et al.

Public-key encryption (PKE) was one of the most fundamental primitives in cryptography. The first security notion for PKE was indistinguishability of encryptions under chosen-plaintext attacks (indistinguishability against chosen-plaintext attacks) (IND-CPA or CPA) (Stinson 2005; Katz and Lindell 2007), also known as semantic security (Stinson 2005; Katz and Lindell 2007). Although CPA security was not stronger than CCA security (Stinson 2005; Katz and Lindell 2007), the research on the cryptosystems which were still CPA secure was significant. For example, it could become a fundamental of the cryptosystems which were CCA-secure (Stinson 2005; Katz and Lindell 2007). In recent years, construction of the lattice-based PKEs had attracted a lot of attention, too. One of the main fields of interest in cryptography was the design and analysis of PKE schemes that were CPA security. However, there were only a handful of known lattice-based PKEs that enjoyed CPA security (Stinson 2005; Katz and Lindell 2007): Regev et al. proposed a lattice-based PKE (Regev 2005) which was CPA-secure; In 2008 and in 2009, Peikert et al. proposed two lattice-based PKEs which were against CPA (Peikert et al. 2008), respectively; Gentry et al. proposed a “*dual*” scheme (Gentry et al. 2008) which was CPA-secure; Gentry et al. presented a BGN-type scheme (Gentry et al. 2010) enjoying CPA security; Lindner and Peikert (2011) proposed a lattice-based PKE with better key size. These above lattice-based PKEs achieved CPA security.

In 2008, Gentry et al. (2008) first presented the definition of Preimage Sampleable Functions, gave the specific structure of the general Inhomogeneous Small Solution (ISIS) hard problem and showed that to solve the average-case ISIS problem (Micciancio and Regev 2007) was at least as hard as to quantumly solve the worst-case hard approximation SIVP problems (Gentry et al. 2008; Micciancio 2011). There were a few of PKE schemes based on SIS (Gentry et al. 2008; Lyubashevsky and Wichs 2015).

In 2014, Wang et al. (2014) first proposed Bilateral Inhomogeneous small integer solution problem (Bi-ISIS) on lattice, computational Bi-ISIS (CBi-ISIS) assumption and decisional Bi-ISIS (DBi-ISIS) assumption. Meanwhile, they constructed a lattice-based KE which relied on DBi-ISIS problem in case of worst-case hardness of lattice problem. But until now, there is no lattice-based PKE on Bi-ISIS (Wang et al. 2014). To deal with the problem, we build a PKE on previous works of Wang et al. (2014) and Regev (2005). We take the first step in this direction by constructing a lattice-based PKE on Bi-ISIS (Wang et al. 2014) and proving its CPA security (Stinson 2005; Katz and Lindell 2007). In addition, we give an extended structure PKE of matrix form with multiple bits that is CPA secure (Stinson 2005; Katz and Lindell 2007).

This paper is organized as follows. “Preliminaries” section contains a few preliminaries necessary for our constructions such as definitions and properties related to lattice and PKE schemes. In “A lattice-based PKE scheme” section, we determine our lattice-based PKEs on DBi-ISIS problem, prove its security against CPA, draw detailed comparisons with related work in the literature and gives a PKE of matrix form which is CPA-secure. In “Conclusion” section, we state conclusion and open problems. Acknowledgements section gives the acknowledgement.

## Preliminaries

**Notations** Assume that *n* is the the main security parameter in this paper. Bold lower-case letters denote vectors in the column form, e.g. **x**. Bold capital letters denote matrix, e.g. **A**, and the transposition of **A** is ${\mathbf{A}}^t$. The Euclidean ($l_2$) norm for vectors, denoted by $\parallel {\mathbf{x}}\parallel_2=\sqrt{\sum_i {x_i^2}}$, is used. That choosing elements from the set *X* uniformly at random is denoted by $x_1,\ldots ,x_k\leftarrow_R X$.

### Hard random integer lattice

Here mainly describe some definitions and properties on lattice (Regev 2005, 2009).

#### **Definition 1**

(Regev 2005) Given *k* linearly independent column vectors ${\mathbf{b}}_1,\ldots ,{\mathbf{b}}_k \in {\mathbb{R}}^n$, let $B=[{\mathbf{b}}_1,\ldots ,{\mathbf{b}}_k]\in R^{n\times k}$ with the basis column vectors, the *n*-dimensional lattice $\Lambda$ with rank$(\Lambda )=k$ in *n* dimensional real space ${\mathbb{R}}^n$ generated by

$$ \varLambda ={\mathcal{L}}(B)=\left\{y\in R^n,\,\hbox {s.t.}\,\exists x=(x_1,\ldots ,x_k,)\in Z^k,\,y=Bx=\sum \limits_{i=1}^k x_i {\mathbf{b}}_i\right\}$$

where *Bx* is the usual matrix-vector multiplication.

#### **Definition 2**

(Regev 2005) For integers $q,\,m>n$, (e.g. $m={O}(n\log n)$, $q=O (n^2))$, ${\mathbf{A}} \in Z_q^{n\times m}$, rank$(A)=n$, defined:

$$ \begin{aligned} \varLambda_q(A)&=\{ \mathbf{v } \in {\mathbb{Z}}^m:\, \mathbf{v }=\mathbf{A }^t \mathbf{z } \mod q,\, \forall z\in {\mathbb{Z}}^n\}\\ \varLambda_q^\bot (A)&=\{\mathbf{v } \in {\mathbb{Z}}^m:\,A\mathbf{v }=0 \mod q\} \end{aligned}$$

The most well-known basic hard computational lattice problems are SVP (Gentry et al. 2008; Micciancio 2011) and CVP (Gentry et al. 2008; Micciancio 2011). And the worst-case problems underlying ocryptosystem are to approximate SIVP or GapSVP (Gentry et al. 2008; Micciancio 2011).

#### **Definition 3**

(SIVP) (Micciancio 2011) Given a lattice basis $B\in Z^{n\times n}$, find *n* linearly independent lattice vectors $S=[s_1, \ldots, s_n ]$ (where $s_i\in {\mathcal{L}}(B)$ for all *i*) minimizing the quantity $\Vert S\Vert = max_i \Vert s_i \Vert$.

The approximation variants of these problems: $GapSVP_\gamma$(Gentry et al. 2008) and $SIVP_\gamma$ (Gentry et al. 2008), which are extension of SVP [$SVP_\gamma$ (Gentry et al. 2008)], are two standard (worst-case hard) approximation problems on lattices, where $\gamma =\gamma (n)$ denotes the approximation factor as a function of the dimension.

#### **Definition 4**

(SVP (Decision Version): $GapSVP_\gamma$) (Gentry et al. 2008) An input to $GapSVP_\gamma$ is basis *B* of a full-rank $n-$demensional lattice. It is a YES instance if $\lambda_1({\mathcal{L}}(B))\le 1$, and is a NO instance if $\lambda_1({\mathcal{L}}(B))>\gamma (n)$. $\lambda_1(\Lambda )$ can be seen in Micciancio and Regev (2007).

#### **Definition 5**

(${SIVP}_\gamma$) (Gentry et al. 2008) An input to ${SIVP}_\gamma$ is an *n*-dimensional lattice basis *B*. The goal is to output a set of *n* linearly independent lattice vectors $S\subset {\mathcal{L}}(B)$, $S=\{s_1,\ldots ,s_n\}$, such as $\Vert S\Vert \le \gamma (n)\cdot \lambda_n({\mathcal{L}}(B))$, $\Vert S\Vert =max_i\Vert s_i\Vert$.

### Hard average-case problems: the small integer solution (SIS)

The hard-on-average problem first proposed by Ajtai (1996) was the SIS problem (Micciancio and Regev 2007) and its variant problem was the inhomogeneous SIS problem (ISIS) (Micciancio and Regev 2007). This was syntactically equivalent to finding some short nonzero vectors in $\Lambda^\bot (A)$ (Regev 2005; Gentry et al. 2008).

#### **Definition 6**

(SIS ($SIS_{q,m,\beta }$)) (Micciancio and Regev 2007): given an integer *q*, a uniformly random $A\in Z_q^{n\times m}$, and a real $\beta$, find a nonzero integer vector $e\in Z^m\backslash \{0\}$ such that $Ae=0\mod q$ and $\Vert e\Vert \le \beta$.

#### **Definition 7**

(ISIS ($ISIS_{q,m,\beta }$)) (Micciancio and Regev 2007): given an integer *q*, a uniformly random matrix $A\in Z_q^{n\times m}$, a uniformly random $u\in Z_q^n$, and a real $\beta$, find an integer vector $e\in Z^m\backslash \{0\}$ such that $Ae = u \mod q$ and $\Vert e\Vert \le \beta$.

For $q(n), m(n), \beta (n)$, $ISIS_{q,m,\beta }$ and $SIS_{ q,m,\beta }$ are the ensembles over instances $(q(n),A,\beta (n))$. Obviously, if $u=0 \mod q$, then $ISIS_{q,m,\beta }$ is $SIS_{q,m,\beta }$.

The SIS and ISIS problems are only meaningful if they admit valid solutions for the particular choices of $q, m,\beta$ such as $\beta \ge \sqrt{m}$ and $m\ge 2n\log q$ (Gentry et al. 2008).

Micciancio and Regev (2007) showed that $SIS_{q,m,\beta }$ and $ISIS_{q,m,\beta }$ were as hard (on the average) as approximating certain worst-case problems on lattices to within small factors (Micciancio and Regev 2007; Gentry et al. 2008).

Wang et al. gave variants of SIS/ISIS problems (Gentry et al. 2008): Bi-SIS/Bi-ISIS (Wang et al. 2014).

#### **Definition 8**

(Bi-ISIS) (Wang et al. 2014) Given an integer *q*, a matrix ${\mathbf{A}} \in Z_q^{m\times m}$ chosen randomly with $rank ({\mathbf{A}} )=n$, two vectors ${\mathbf{u}}_1,\,{\mathbf{u}}_2\in {\mathbb{Z}}_q^m$ and a real $\beta$, the goal is to find nonzero integer vectors $x,\,y \in {\mathbb{Z}}^m\backslash \{0\}$ such that

$$ \left\{ \begin{array}{c} {\mathbf{Ax}} ={\mathbf{u}}_1 \mod q,\,\parallel {\mathbf{x}} \parallel \le \beta \\ {\mathbf{y}}^t{\mathbf{A}} ={\mathbf{u}}_2^t \mod q,\,\parallel {\mathbf{y}} \parallel \le \beta \end{array} \right.$$

If $u_1=0 \mod q,\,u_2^t=0 \mod q$, *Bi*-*ISIS* is the *Bi*-*SIS*. $Bi-SIS_{q,m,\beta }/Bi-ISIS_{q,m,\beta }$ denote the probability ensembles over *Bi*-*SIS*/*Bi*-*ISIS* instances. Lemma 9 and Proposition 10 (Wang et al. 2014) gave the hardness of $Bi-SIS_{q,m,\beta }$ and $Bi-ISIS_{q,m,\beta }$.

#### **Lemma 9**

(Wang et al. 2014) *The problems*$Bi-SIS_{q,m,\beta }/Bi-ISIS_{q,m,\beta }$*are as hard as the problems*$SIS_{q,m,\beta }/ISIS_{q,m,\beta }$, *respectively*.

#### **Proposition 10**

(Wang et al. 2014) *Given any poly-boundedm*, $\beta =poly(n)$, $q\ge \beta \cdot \omega (\sqrt{n\log n}$), *the*$Bi-SIS_{q,m,\beta }$*and*$Bi-ISIS_{q,m,\beta }$*problems in average case are as hard as approximating the problem*$SIVP_\gamma$*and GapSVP, in the worst case within certain*$\gamma =\beta \cdot \tilde{O}(\sqrt{n})$.

#### **Definition 11**

(${Bi-ISIS}^*$) (Wang et al. 2014) Let *n*, *m*, *q* and $\beta$ be the parameters as that of *ISIS*, ${\mathbf{A}} \in Z_q^{m\times m}$ with rank(${\mathbf{A}} )=n$, ${\mathbf{e}}_1$ is linear independent with column vectors of **A**, ${\mathbf{e}}_2$ is linear independent with row vectors of **A**. For vectors

$$\begin{aligned} \mathbf{b }_1\in & {} \{\mathbf{Az }+\mathbf{e }_1:\,\mathbf{z }\in {\mathbb{Z}}^m,\,\mathbf{e }_2^t\cdot \mathbf{z }=0 \mod q\}\\ \mathbf{b }_2^t\in & {} \{\mathbf{z }^t\mathbf{A }+\mathbf{e }_2^t:\,\mathbf{z }\in {\mathbb{Z}}^m,\,\mathbf{z }^t \cdot \mathbf{e }_1=0 \mod q\} \end{aligned}$$

the goal is to find a vector $x\in {\mathbb{Z}}^m$ and a vector $y\in {\mathbb{Z}}^m$ s.t.

$$\left\{ \begin{array}{c} {\mathbf{Ax}} +{\mathbf{e}}_1={\mathbf{b}}_1 \mod q,\,\parallel {\mathbf{x}} \parallel \le \beta \\ {\mathbf{y}}^t{\mathbf{A}} +{\mathbf{e}}_2^t={\mathbf{b}}_2^t \mod q,\,\parallel {\mathbf{y}} \parallel \le \beta \end{array} \right.$$

If $e_1,\,e_2$ are unknown, ${Bi-ISIS^*}$ is much harder than *Bi*-*ISIS*.

Now we give the formulas of CBi-ISIS/DBi-ISIS problem (Wang et al. 2014). Here we only give definition of DBi-ISIS problem and DBi-ISIS assumption, CBi-ISIS problem and CBi-ISIS assumption were in Wang et al. (2014).

Given security parameters *n*, *q*, *m*, $\beta$, a matrix ${\mathbf{A}} \in {\mathbb{Z}}_q^{m\times m}$ with rank(*A*) = n. Let $D=\{z\in Z^m:\,\parallel z \parallel_2\le \beta \}$. For any vectors $x\in D$ and $y\in D$, there exist two vectors sets $\mathbf{U }=\{\mathbf{u }_1,\ldots ,\, \mathbf{u }_n\}$, which is linear independent with the column vectors of **A**, and $\mathbf{V }=\{\mathbf{v }_1,\ldots ,\,\mathbf{v }_n\}$ which is linear independent with the row vectors of **A**, such that for $\forall i\in \{1,\ldots ,n\}$, $y^t\cdot u_i=0 \mod q$, $v_i^t\cdot x=0 \mod q$. Denote them by notations:

$$ A*x:=Ax+\sum \limits_{i\in S}u_i \mod q,\quad y^t * A:=y^t A +\sum \limits_{i\in S'}v_i^t \mod q$$

where *S* and $S^{\prime }$ are two random subsets of $\{1,\ldots ,\,n\}$.

#### **Definition 12**

(DBi-ISIS problem) (Wang et al. 2014) Given $(A,A*x,y^t*A,y^tAx)$, the goal is to distinguish $(A,A*x,y^t*A,y^tAx)$ and $(A,A*x,y^t*A,z)$, where $x,\,y\in D$ and $z\in {\mathbb{Z}}_q$ are chosen uniformly at random.

#### **Definition 13**

(DBi-ISIS assumption) (Wang et al. 2014) Let $n,\,m=poly(n)$ be integers, $m>n$, and $\beta =poly(n)$ be a real such that $q\ge \beta \cdot \omega \sqrt{(n\log n)}$ and $D=\{z\in Z^m:\,\parallel z \parallel_2\le \beta \}$, $A\in Z_q^{m\times m}$ be a random matrix with rank$(A)=n$. Then for any probabilistic polynomial time (PPT) ${\mathcal{A}}$, the following holds:

$$ \begin{aligned}&Pr[{\mathcal{A}}(A,\beta ,A*x,y^t*A,y^tAx)=1:x,y\leftarrow_R D]\\&-Pr[{\mathcal{A}}(A,\beta ,A*x,y^t*A,z)=1:x,y\leftarrow_R D]|<negl(n) \end{aligned}$$

where the probability is taken over the random choice of $x,\,y,\,z\leftarrow_R D$ and the random bits used by ${\mathcal{A}}$.

A PKE scheme is a tuple of PPT algorithms $\prod =(KeyGen, Encrypt, Decrypt)$ (or $\prod = (Gen,Enc,Dec)$) (Katz and Lindell 2007). Here consider the experiment defined for PKE $\prod =(Gen,Enc,Dec)$ and an adversary ${\mathcal{A}}$. The CPA indistinguishability experiment is $PubK_{{\mathcal{A}},\prod }^{cpa}(n)$. $PubK_{{\mathcal{A}},\prod }^{cpa}(n)=1$ stands for the probability of ${\mathcal{A}}$ attacking experiment $\prod$ successfully (Katz and Lindell 2007). (In case $PubK_{{\mathcal{A}},\prod }^{cpa}(n)=1$, say ${\mathcal{A}}$ succeeds (Katz and Lindell 2007)).

#### **Definition 14**

(Katz and Lindell 2007) A PKE scheme $\prod =(Gen,Enc,Dec)$ has *indistinguishable encryptions under CPA* (CPA security) if for all PPT adversaries ${\mathcal{A}}$, there exists a negligible function *negl* such that:

$$ Pr[PubK_{{\mathcal{A}},\prod }^{cpa}(n)=1]\leqslant \frac{1}{2}+negl(n)$$

### Security model for CPA 

We briefly review the notion of CPA security (Katz and Lindell 2007) which is defined using the game between a challenger and an adversary ${\mathcal{A}}$. Both are given the security parameter $1^n$ as input. Specifically, set a PKE experiment $\prod =(Gen, Enc, Dec)$ and an adversary ${\mathcal{A}}$, the CPA experiment is $PubK_{{\mathcal{A}},\prod }^{cpa}(n)$ as follows.

*Setup* The challenger runs $KeyGen(1^n)$ to get a pair of public and private key (*pk*, *sk*). The challenger gives ${\mathcal{A}}$*pk* as well as oracle access to $Enc_{pk}(\cdot )$ and keeps *sk* private.

*Queries phase 1*${\mathcal{A}}$ can issue encryption queries *m* where message *m* must be in the plaintext space associated with *pk*. The challenger responds with *Encrypt*(*pk*, *m*) (or $Enc_{pk}(m)$).

*Challenge*$${\mathcal{A}}$$ outputs two messages $$m_0$$ and $$m_1$$ of equal length to challenger. The challenger picks $$b\in \{0,1\}$$ at random and encrypts $$ m_b$$ to get challenge ciphertext $$C=Encrypt(m_b,pk)$$ (or $$C=Enc_{pk}(m_b)$$). The challenger gives *C* to $${\mathcal{A}}$$.

*Queries phase 2*${\mathcal{A}}$ continues to have access to $Enc_{pk}(\cdot )$ oracle and issues encryption queries *m* as in phase 1, with the added constraint that $m\ne m_b$. The challenger responds with *Encrypt*(*pk*, *m*) ($Enc_{pk}(m)$).

*Guess*${\mathcal{A}}$ outputs its guess (a bit) $b'\in \{0,1\}$ of *b*. If $b' = b$, the simulator outputs 1 (indicating that ${\mathcal{A}}$ wins the game (experiment)); otherwise the simulator outputs 0. And the output of the experiment is defined to be 1 if $b' = b$, and 0 otherwise. (In case $PubK^{cpa}_{{\mathcal{A}},\prod }(n)=1$, we say that ${\mathcal{A}}$ succeeds.)

Define the advantage of ${\mathcal{A}}$ in this game as $Adv_{{\mathcal{A}},\prod }^{cpa}(n)=|Pr[b'=b]-\frac{1}{2}|$. A PKE is CPA-secure if no PPT adversaries ${\mathcal{A}}$ have non-negligible advantage in this CPA game.

### Select parameters

Here the parameters are chosen the same as that in Wang et al. (2014): $q=O(n^2)$ is prime, $m=O(n\log n),\,\beta \ge \sqrt{m}$, $q/\omega (\sqrt{n\log n})> \beta \ge \sqrt{m}$, and $m \ge 2n\log q$, e.g. for the typical parameters $q=n^2$, $m=2n\log q$, and $\beta =\sqrt{m}=2\sqrt{n\log n}.$

## A lattice-based PKE scheme

In this section, we give a direct construction of a CPA-secure PKE scheme under DBi-ISIS assumption: a simple lattice-based PKE scheme.

### The encryption scheme

In this subsection, we present the full description of our PKE scheme.

*Setup* Let *n* be the security parameter that is parameterized by three integers, $m=m(n),\,q=q(n)$, a real number $\beta =\beta (n)$, $A\in Z_q^{m\times m}$ with $rank(A)=n,\,m>n$. All computing is performed in $Z_q$, e.g. modulus *q*.

*Initialize* Given a public matrix $A\leftarrow_R Z_q^{m\times m}$ with $rank(A)=n$, a short vector set $D=\{z\in Z^m:\, \parallel Z\parallel \le \beta \}$. Generate $V=\{v_1^t,\ldots ,v_n^t\}$ which is linear independent with row vectors of *A*, $U=\{u_1,\ldots ,u_n\}$ which is linear independent with column vectors of *A*, and make *V*, *U* public.

*KeyGen*($1^n$) Let *y* be the secret key, and $p_B=y^t*A=y^tA+\sum \nolimits_{i=1}^n v_i^t \mod q$ be the public key (which is used to encrypt the plaintext), where $y\leftarrow_R D$ such that $\langle u_i,y\rangle=0 \mod q$.

*Encrypt*($p_B, m$) To encrypt a message $m\in Z_q$, first pick a random vector $x\leftarrow_R D$ such that $\langle v_i,x \rangle=0 \mod q$ (*x* can be generated by pseudorandom generators). Then compute

$$ C_1=A*x=Ax+\sum \limits_{i=1}^n u_i\mod q,\quad C_2=m+p_Bx \mod q $$

The ciphertext is $C=(C_1,C_2)$. Erase *x* secretly (if the sender and the receiver are at the same places, erase *x* directly) and output ciphertext *C*.

*Decrypt*($C=(C_1,C_2), y$) To decrypt the ciphertext $C=(C_1,C_2)$ with the private key $y^t$, first calculate $K=y^t \cdot C_1 \mod q$. Then decrypt finishes as follows $m=C_2-K \mod q$.

*Correctness* If the PKE is run honestly, then *m* can be obtained successfully such that $<v_i,x>=0 \mod q,\, <u_i,y>=0 \mod q$.

The scheme’s correctness (with overwhelming probability) follows by the form of $C_1,\,C_2,\,K$:

$$ \begin{aligned} C_1&=A*x=Ax+\sum \limits_{i=1}^n u_i \mod q\\ C_2&=m+p_B\cdot x=m+\left( y^tA+\sum \limits_{i=1}^n v_i^t\right) \cdot x\\&=m+y^tA\cdot x+\sum \limits_{i=1}^n v_i^tx=m+y^tA\cdot x \mod q\\ K&=y^t \cdot C_1=y^t\cdot \left( Ax+\sum \limits_{i=1}^n u_i\right) \\&=y^t\cdot Ax+y^t\cdot \sum \limits_{i=1}^n u_i =y^t\cdot Ax \mod q \end{aligned} $$

with $\langle v_i,x \rangle=0 \mod q, \langle u_i,y \rangle=0 \mod q$, we have that

$$ C_2-K=m+y^t\cdot Ax-y^t\cdot Ax=m \mod q$$

### Security under CPA

We utilize the “Game hopping” (Dent 2006) to prove its CPA security of our lattice-based PKE scheme. We reduce its CPA security to the DBi-ISIS assumption. If a PPT adversary ${\mathcal{A}}$ wins the CPA game with non-negligible advantage, then we can construct a simulator that distinguishes a DBi-ISIS tuple from a random tuple with non-negligible advantage.

#### **Theorem 15**

*IfDBi*-*ISISproblem is hard for a PPT algorithm*${\mathcal{G}}$, *then the lattice-based PKE scheme in* “The encryption scheme” *section has indistinguishable encryptions under CPA. Namely, our PKE shcheme is CPA secure underDBi*-*ISISassumption*.

#### *Proof*

Suppose there exists an adversary ${\mathcal{A}}$ (or an algorithm) to win the CPA game, we build a simulator (an algorithm), ${\mathcal{S}}$, that has non-negligible advantage in solving DBi-ISIS problem.

Let $\prod$ be the lattice-based PKE scheme in “The encryption scheme” section. Suppose that ${\mathcal{A}}$ is a PPT adversary, and define

$$ \varepsilon (n)=Pr[PubK_{{\mathcal{A}},\prod }^{cpa}(n)=1] $$

Let $\tilde{\prod }$ be the modified PKE, where *GenKey* is the same as in $\prod$. But to encrypt a message $m\in Z_q$ with public key $(Z,q,A,P_B)$, the sender selects $x, z_1,z_2\leftarrow_R D$ and computes ciphertext $C=(C_1,C_2)=(A*x,(z_2^t*A)z_1+m)$.

Although the receiver cannot calculate the plaintext *m* from $\tilde{\prod }$, $PubK_{{\mathcal{A}},\tilde{\prod }}^{cpa}(n)$ is still well-defined since the experiment depends only on $KeyGen(\cdot )$, $encrypt(\cdot )$.

Now we discuss that the ciphertext in $\tilde{\prod }$ is independent of the plaintext *m* being encrypted. Virtually, when $z_1,\,z_2\leftarrow_R D$, $z_2^t(A*z_1)\mod q$ and $(z_2^t*A)z_1\mod q$ are two random elements in $Z_q$. This implies that $m+z_2^t(A*z_1)\mod q,\,m+(z_2^t*A)z_1\mod q$ are independent of *m*. Obviously, the first element $C_1=y^t*A \mod q$ in $\tilde{\prod }$ has no relationship with plaintext *m*. Taken together, the ciphertext in $\tilde{\prod }$ is independent of *m* and hence contains no information about *m*. Thus we get

$$ Pr\left[ PubK_{{\mathcal{A}},\tilde{\prod }}^{cpa}(n)=1\right] =\frac{1}{2}$$

The simulator ${\mathcal{S}}$ now plays the role of challenger in the CPA game and tries to solve DBi-ISIS problem (we recall that when ${\mathcal{S}}$ receives ($Z_q,Z_q^m,A,q,P_B, C_1,C_3$) where for $\forall x,\,y,\,z_1,\,z_2\leftarrow_R D$

$$ C_1=A*x=Ax+\sum \limits_{i=1}^n u_i \mod q,\,p_B=y^t*A=y^tA+\sum \limits_{i=1}^n v_i^t \mod q$$

$C_3$ is equal to $(y^t*A)x$ or $C_3$ is equal to $(z_2^t*A)\cdot z_1$ such that $\langle z_2,u_i \rangle=0\mod q, \langle z_1,v_i \rangle=0\mod q$). The simulator ${\mathcal{S}}$ that accomplishes this simulates the view of the adversary ${\mathcal{A}}$ as follows.

${\mathcal{S}}$ takes $Z_q,q,A,C_1,C_2,C_3$ as input.

*Setup* The adversary ${\mathcal{A}}$ is given the public key $A,\, p_B=y^t*A=y^tA+\sum \nolimits_{i=1}^n v_i^t \mod q$ whose corresponding private key is $y\leftarrow_R D$ such that $\langle y,u_i \rangle =0\mod q$. (Here $A,\, p_B$ are used to encrypt the message *m*.)

*Queries phase 1* The adversary ${\mathcal{A}}$ issues encryption queries. The adversary has unlimited access to $Enc_{p_B}(\cdot )$ oracle with input a message *m*, where *m* is an alleged plaintext.

*Challenge* The adversary ${\mathcal{A}}$ submits two messages $m_0$ and $m_1$ and sends them to the simulator. The simulator ${\mathcal{S}}$ flips a coin, *b*, constructs the challenge ciphertext $C=(C_1, C_2)$ of $m_b,\,b\in \{0,1\}$ and gives it to ${\mathcal{A}}$, where $C_1=A*x \mod q$, $C_2=C_3+m_b\mod q$.

We note that *C* is a valid encryption of $m_b$ if the simulator ${\mathcal{S}}$ is given a DBi-ISIS tuple. Otherwise, if ${\mathcal{S}}$ is given a random tuple, *C* is independent of *b* in the ${\mathcal{A}}'s$ view.

*Queries phase 2* Same as phase 1. But the adversary is not allowed to query the $Enc_{p_B}(\cdot )$ oracle on message $m_b$.

*Guess* Then $${\mathcal{A}}$$ continues to have oracle access to $$Enc_{p_B}(\cdot )$$ and outputs its guess $$b'$$ of *b*. If $$b'=b$$, $${\mathcal{S}}$$ outputs 1 and answers “DBi-ISIS” (indicating that $$C_3=y^tAx)$$; otherwise $${\mathcal{S}}$$ outputs 0 and answers “random” (indicating that $$C_3=z_2^tAz_1$$). More precisely, there are two corresponding cases of $${\mathcal{S}}'$$s performances. (The simulator $${\mathcal{S}}$$ takes as input a random challenge $$C=(C_1,C_3+m_b)$$ where $$C_3$$ is either $$y^tAx$$ or a random element of $$Z_q$$.) The simulator $${\mathcal{S}}$$ proceeds as follows.

1. ${\mathcal{S}}$ runs ${\mathcal{G}}(1^n)$ to get $(Z_q,Z_q^m,A,q)$ which can be regarded as ${\mathcal{S}}'s$ input. ${\mathcal{S}}$ chooses $x,\,y,\,z_1,\,z_2\leftarrow_R D$ and sets
  $$ \begin{aligned} C_1&=A*x=Ax+\sum \limits_{i=1}^n u_i \mod q\\ p_B&=y^t*A=y^tA+\sum \limits_{i=1}^n v_i^t \mod q\\ C_3&=(z_2^t*A)\cdot z_1\mod q\\ (C_3&=z_2^t\cdot (A* z_1)\mod q) \end{aligned} $$
  Then ${\mathcal{S}}$ runs algorithm ${\mathcal{A}}$ on a public key constructed as
  $$ pk= \langle Z_q,Z_q^m,A,q,p_B \rangle $$
  and a ciphertext constructed as
  $$ C=(C_1,C_2)=(A*x,C_3+m_b)$$
  In this case, $C_3=(z_2^t*A)\cdot z_1\mod q$ (or $C_3=z_2^t\cdot (A* z_1)\mod q$), Thus we have that $C_2$ is completely random from the view of ${\mathcal{A}}$, which implies that ${\mathcal{A}}'$s view is the same as ${\mathcal{A}}'$s view in $PubK_{{\mathcal{A}},\tilde{\prod }}^{cpa}(n)$ since *C* is completely random. Because ${\mathcal{S}}$ outputs 1 when ${\mathcal{A}}$ outputs $b'$: $b'=b$, hence
  $$ Pr[{\mathcal{S}}(Z_q,z_q^m,A,q,y^t*A,A*x,(z_2^t*A)\cdot z_1)=1] =Pr\left[ PubK_{{\mathcal{A}},\tilde{\prod }}^{cpa}(n)=1\right] =\frac{1}{2}$$
2. ${\mathcal{S}}$ runs ${\mathcal{G}}(1^n)$ to obtain $(Z_q,Z_q^m,A,q)$ which can be regarded as the input of ${\mathcal{S}}$. Then, ${\mathcal{S}}$ selects $x,\,y\leftarrow_R D$, sets

$$ \begin{aligned} C_1&=A*x=Ax+\sum \limits_{i=1}^n u_i \mod q\\ p_B&=y^t*A=y^tA+\sum \limits_{i=1}^n v_i^t \mod q\\ C_3&=(y^t*A)x \mod q \end{aligned}$$

Finally, ${\mathcal{S}}$ runs algorithm ${\mathcal{A}}$ on a public key constructed as

$$ pk=(Z_q,z_q^m,A,q,P_B) $$

and a cipherext constructed as

$$ C=(C_1,C_2)=(A*x,y^tAx+m_b)=(A*x,(y^t*A)x+m_b)$$

Obviously, in this case, $C_3=(y^t*A)x \mod q$, then $C_2$ is a valid ciphertext. That means that ${\mathcal{A}}'s$ view distribution is exactly as ${\mathcal{A}}'s$ view in $PubK_{{\mathcal{A}},\prod }^{cpa}(n)$ since *C* is a valid ciphertext. ${\mathcal{S}}$ outputs 1 when ${\mathcal{A}}$ outputs $b'$: $b'=b$, hence

$$ Pr[{\mathcal{S}}(Z_q,z_q^m,A,q,y^t*A,A*x,y^tAx)=1]= Pr[PubK_{{\mathcal{A}},\prod }^{cpa}(n)=1]=\varepsilon (n) $$

We see that if $C_3$ is sampled from random, $C=(C_1,C_2)$ is random; if $C_3$ is sampled from *DBi*-*ISIS* game, $C=(C_1,C_2)$ is the valid ciphertext. Putting together the two cases, it follows that ${\mathcal{A}}'$s advantage $Adv_{{\mathcal{A}},\prod }^{cpa}(n)$ in distinguishing between the real “DBi-ISIS” and “random” is negligibly close to

$$ \begin{aligned} |&Pr[{\mathcal{S}}(Z_q,z_q^m,A,q,y^t*A,A*x,(z_2^t*A)z_1)=1]\\&-Pr[{\mathcal{S}}(Z_q,z_q^m,A,q,y^t*A,A*x,y^TAx)=1]| =\left|\frac{1}{2}-\varepsilon (n)\right| \end{aligned}$$

that is $Adv_{{\mathcal{A}},\prod }^{cpa}(n)=|Pr[PubK_{{\mathcal{A}}, \prod }^{cpa}(n)=1]-\frac{1}{2}|$. Since the *DBi*-*ISIS* problem is hard, there must exist a negligible function *negl*(*n*) such that

$$\begin{aligned}&negl(n)\ge | Pr[{\mathcal{S}}(Z_q,z_q^m,A,q,y^t*A,A*x,(z_2^t*A)z_1)=1]\\&-Pr[{\mathcal{S}}(Z_q,z_q^m,A,q,y^t*A,A*x,y^TAx)=1]|=\left|\frac{1}{2}-\varepsilon (n)\right| \end{aligned}$$

which implies that $\varepsilon (n)\le \frac{1}{2}+negl(n)$ (In other words, *S* has advantage at most *negl*(*n*) in solving DBi-ISIS problem). It follows that our PKE in “The encryption scheme” section *has indistinguishable encryptions under CPA*. By Definition 14, we have

$$ Pr[PubK_{A,\prod }^{cpa}(n)=1]\le \frac{1}{2}+negl(n)$$

All in all, for all PPT adversaries ${\mathcal{A}}$, the lattice-based PKE system in “The encryption scheme” section is CPA security if the DBi-problem is hard. This completes the proof. $\square$

###  Comparison with lattice-based PKEs

**Table 1:** Comparisons with lattice-based cryptosystems

Pub	*Pub.size*	*Cipher.size*	*Enc.Comp.*	*Dec.Comp.*	*Priv.size*	*Plaint.size*
Regev (2005)	$2(n+1)n\log^2q$	$n\log^2q+\log q$	$2n^2\log q$	*n*	$n\log q$	1
Lindner and Peikert (2011)	$$2n^2\log q$$	$$2n\log q $$	$$3n^2 $$	$$n^2$$	$$n^2\log q$$	$$n \log q $$
Gentry et al. (2010)	$2n^2log^2q$	$4n^2log^3q$	$4n^2log^2q(n+1)$	$8n^3log^3q(1+24n^3log^3q)$	$4n^2log^3q$	$4n^2log^2q$
Ours	$2n\log^2 q(2n\log q+1)$	$2n\log^2 q+\log q$	$2n\log q(2n\log q+1)$	$2n\log q$	$2n\log^2 q$	$\log q$

For the comparison to be meaningful, we consider the latticed-based PKEs. Table 1 shows the comparison in the term of communication complexity (complexity of space or storage efficiency, e.g. *Pub.size*) and computation complexity (e.g *Enc.comp.*) of PKEs. *Pub.size* means the size of the public key, others are so; *Comput.Comp.* means the computation complexity and is estimated by the number of the multiplications in $Z_q$. Their main computation operation contains the matrix-vector multiplication in $Z_q$. Table 1 shows the comparison in more detail. Here *n* is the security parameter, *q* is a polynomial function of *n*.

Compared with PKEs (Regev 2005; Lindner and Peikert 2011), from the respective of the space complexity of view, *priv.size* in our system is nearly the same as that of Regev (2005) but smaller than that in Lindner and Peikert (2011); the public key size is slightly bigger than that of Regev (2005) and Lindner and Peikert (2011); the *Cipher.size* is almost the same as that of Regev (2005) and Lindner and Peikert (2011); and the range of the encrypted plaintext is significantly bigger than that in Regev (2005) but smaller than that of Lindner and Peikert (2011). As for *Comput.comp*, our *Enc.comp* is a little bigger than that of Regev (2005) and Lindner and Peikert (2011); our *Dec.comp* is nearly identical to that in Regev (2005), but smaller than that in Lindner and Peikert (2011). Compared with the BGN-type PKE (Gentry et al. 2010), the performance of our PKE is much better except for *Plain.size* and *Pub.size*. Obviously, *Cipher.size*, *Enc.Comp.*, *Dec.Com.*, *Priv.size* and *Plain.size* are all much smaller than that of Gentry et al. (2010) although *Pub.size* of Gentry et al. (2010) is a little smaller than our *Pub.size*.

In short, our lattice-based scheme is equally advantageous to the scheme in Regev (2005) except for the *plain.size*. Our scheme enjoys almost the same advantages as that in Lindner and Peikert (2011) in the aspects of *Pub.size*, *Cipher.size*, *Enc.Com.*, but *Dec.comp* and *Priv.size* are much more advantageous than that in Lindner and Peikert (2011), *plain.size* is smaller. But our scheme has more advantages than that of Gentry et al. (2010) in all aspects which are mentioned in Table 1 except for *Plain.size*, *Pub.size*. In addition, all the PKEs of Regev (2005), Lindner and Peikert (2011) and Gentry et al. (2010) are from the LWE problem while ours depends on the SIS problem. The PKEs of Regev (2005), Lindner and Peikert (2011) and Gentry et al. (2010) and ours are all CPA-sure and resist quantum attack.

### A lattice-based extended structure PKE of matrix form

To sent plaintext with multiple bits in our PKE, one can use matrix secret and matrix plaintext. Now we describe the PKE in “multiple bits” scenario and show its CPA security underlying DBi-ISIS assumption.

#### Basic construction

In the following, we convert the lattice-based KE on SIS (Wang et al. 2014) into an asymmetric PKE with multiple bits: *an extended structure PKE of matrix form*.

Use the same parameters $m=m(n),\,q=q(n),\,\beta =\beta (n)$ and $A\in Z_q^{m\times m}$ with $rank(A)=n,\,n< m$ as mentioned above.

*Setup* Generate public parameters $$n,\,m,\,n<m$$, a real number $$\beta $$, a prime *q*, a random matrix $$A\in Z_q^{m\times m}$$ with $$rank(A)=n $$, a short vector set $$D=\{z\in Z^m:\, \parallel Z\parallel \le \beta \}$$.

*Initialize* Assume that pick randomly $X\leftarrow_R D^{k_1}$, generate vector group *V* which are linearly independent with row vectors of *A*, such that $V^t\cdot X=0 \mod q$, then keep *X* private and make *V* public. Suppose that randomly pick $Y\leftarrow_R D^{k_2}$, choose vector group *U* which are linearly independent with column vectors of *A*, such that $Y^t U=0\mod q$, then keep *Y* private and make *U* public, where $k_1,k_2$ are integers.

*KeyGen* Let

$$ P_B=Y^t*A=Y^tA+V^t\in Z_q^{k_2\times m} \mod q$$

be the public key which is used to encrypt message, $Y^t$ is the corresponding private key.

*Encrypt* To send a message $M\in Z_q^{k_2\times k_1}$, pick $X\leftarrow_R D^{k_1}$ as a random value such that $V^t\cdot X=0 \mod q$ (*X* can be generated by the pseudorandom generator. If the sender and the receiver are at the same local, *X* is deleted once the ciphertext is completed successfully).

Then compute

$$ \begin{aligned} C_1&=A*X=AX+U\mod q\\ C_2&=M+P_BX\mod q \end{aligned}$$

Output ciphertext $C=(C_1,C_2)$ and erase *X* secretly.

*Decrypt* Upon receiving $C=(C_1,C_2)$, knowing the private key $Y^t$, decrypt the message by first calculating

$$ K=Y^t \cdot C_1 \mod q $$

Then compute $C_2-K \mod q$ to get message *M*.

*Correctness* If the system is run honestly, the message *M* is obtained correctly.

To show the correctness of our scheme, $C_1,\,C_2,\,K$ can be written as follows:

$$ \begin{aligned} C_1&=A*X=AX+U \mod q\\ C_2&=M+P_BX=M+(Y^tA+V^t)X\\&=M+Y^tAX+V^tX\mod q\\ K&=Y^t \cdot C_1=Y^t\cdot (AX+U)\\&=Y^t\cdot AX+Y^tU \mod q \end{aligned}$$

then we obtain message *M* by computing

$$C_2-K=M+Y^tAX+V^tX-(Y^t\cdot AX+Y^tU)=M\mod q$$

such that $$V^t\cdot X=0 \mod q$$, $$Y^t U=0\mod q$$.

#### CPA security

Theorem 16 indicates that our extended PKE of matrix form in “Basic construction” section is CPA secure under DBi-ISIS assumption.

##### **Theorem 16**

*If DBi-ISIS problem is hard for a PPT algorithm*${\mathcal{G}}$, *then the lattice-based PKE in* “Basic construction” *section is security against CPA under DBi*-*ISISassumption*.

##### *Proof*

The proof of Theorem 16 is similar to that of Theorem 15, omit it here.

## Conclusion

In this paper, we present a simple PKE scheme that achieves CPA security under the DBi-ISIS assumption. We build it on previous works of Wang et al. (2014) and Regev (2005) and believe that it is easy to understand. Table 1 gives some comparisons with other lattice-based PKEs which indicates that the advantages of our lattice-based PKE are nearly the same as that in Regev (2005), a little different from that of Lindner and Peikert (2011), but much more than that in Gentry et al. (2010).

In addition, we extend the lattice-based PKE in “The encryption scheme” section to a lattice-based extended structure PKE of matrix form with multiple bits in “Basic construction” section indicate its CPA secure.

Note that our PKE schemes in both “The encryption scheme” section and “Basic construction” section may be modified to be security against chosen-ciphertext attacks (CCA) (Stinson 2005; Katz and Lindell 2007). How to improve our proposed PKE schemes to interactive multiparty PKE schemes, how to modify our PKE schemes into signatures based on Bi-ISIS, et al are also worth considering. Maybe our construction is a foundation for other cryptographic primitives constructed. And our construction may be an important step in showing how versatile the SIS assumption can be. We leave them as open problems.
